# Functional Analysis of the Fusarielin Biosynthetic Gene Cluster

**DOI:** 10.3390/molecules21121710

**Published:** 2016-12-13

**Authors:** Aida Droce, Wagma Saei, Simon Hartung Jørgensen, Reinhard Wimmer, Henriette Giese, Rasmus Dam Wollenberg, Teis Esben Sondergaard, Jens Laurids Sørensen

**Affiliations:** 1 Department of Chemistry and Bioscience, Aalborg University, Fredrik Bajers Vej 7H, DK-9220 Aalborg Ø, Denmark; aidadroce@gmail.com (A.D.); wagmasaei@gmail.com (W.S.); sijoer89@gmail.com (S.H.J.); rw@bio.aau.dk (R.W.); hgiese@bio.aau.dk (H.G.); rwo@bio.aau.dk (R.D.W.); tes@bio.aau.dk (T.E.S.); 2 Department of Chemistry and Bioscience, Aalborg University, Niels Bohrs Vej 8, 6700 Esbjerg, Denmark

**Keywords:** *Fusarium graminearum*, trans-enoyl reductase, decalin, polyketide synthases, PKS, secondary metabolites, overexpression, transcription factor, biosynthetic pathway

## Abstract

Fusarielins are polyketides with a decalin core produced by various species of *Aspergillus* and *Fusarium*. Although the responsible gene cluster has been identified, the biosynthetic pathway remains to be elucidated. In the present study, members of the gene cluster were deleted individually in a *Fusarium graminearum* strain overexpressing the local transcription factor. The results suggest that a *trans*-acting enoyl reductase (FSL5) assists the polyketide synthase FSL1 in biosynthesis of a polyketide product, which is released by hydrolysis by a *trans*-acting thioesterase (FSL2). Deletion of the epimerase (FSL3) resulted in accumulation of an unstable compound, which could be the released product. A novel compound, named prefusarielin, accumulated in the deletion mutant of the cytochrome P450 monooxygenase FSL4. Unlike the known fusarielins from *Fusarium*, this compound does not contain oxygenized decalin rings, suggesting that FSL4 is responsible for the oxygenation.

## 1. Introduction

**Figure 1:**
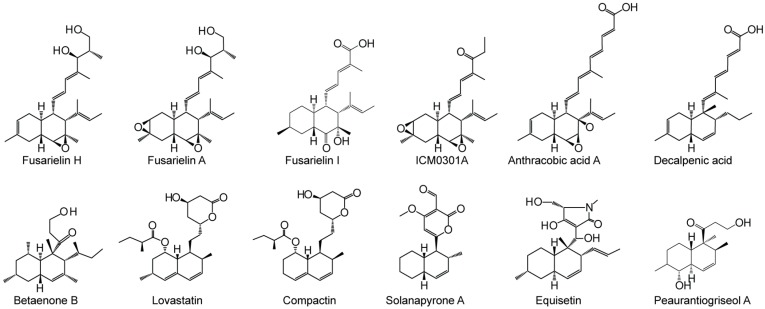
Structure of fusarielins and related decalin polyketides.

Filamentous fungi are a rich source of bioactive natural products and compounds containing a bicyclic decalin ring motif (Figure 1), such as the monacolins lovastatin and compactin. They are of great interest due to their diverse bioactive properties as antibiotics and immunosuppressives [1]. The decalin core is hypothesized to result from an enzymatically guided Diels-Alder cycloaddition event, although only a limiting number of possible Diels-Alderases have been identified [2]. There seems however to be several mechanisms by which Diels-Alder reactions can occur. In the lovastatin biosynthesis, the Diels-Alder reaction occurs at the hexaketide stage and is catalyzed by the highly reducing polyketide synthase (HR-PKS) LovB [3]. In the biosynthesis of solanapyrones, the Diels-Alder reaction is not catalyzed by the PKS (Sol1), which instead releases the pyrone product desmethylprosolanapyrone I [4]. The Diels-Alder reaction is then subsequently catalyzed by a flavin-dependent oxidase (Sol5) [4]. Recently, a protein, Fsa2, without known functional domains was shown to be involved in the control of stereochemistry during the Diels-Alder reaction in the equisetin biosynthetic pathway [5].

Reduction of the growing polyketide chain, before the Diels-Alder reaction, can be mediated by the PKS’s using the β-ketoreductase (KR), dehydratase (DH), and enoyl reductase (ER) [4] as observed in solanapyrones biosynthesis. Some PKS’s, however, have an inactive ER domain; thus, final reduction of carbon–carbon double bonds along the carbon skeleton are achieved though collaboration with a *trans*-acting ER as observed in the biosynthesis of lovastatin [6,7], betaenone [8], and equisetin [9]. After decalin formation and subsequent chain elongation and modification, the resulting decalin polyketide product can be released from the PKS by various mechanisms. A terminal PKS reduction domain catalyzes release in the betaenone and equisetin biosynthesis pathway [8,9], while a *trans*-acting thioesterase (LovG) is involved in the lovastatin pathway [10].

To gain further insight into the biosynthesis of the decalin polyketides, we examined the fusarielin biosynthetic pathway in *Fusarium graminearum*. Fusarielins are derived from the decaketide pathway with five methylations and a decalin core [11]. Fusarielins A–D were first isolated from an unidentified *Fusarium* strain in a selection procedure for antifungal compounds [11]. Later, fusarielins F, G, and H were isolated from *F. graminearum* through constitutive expression of the local transcription factor from the responsible gene cluster [12].

## 2. Results and Discussion

The putative fusarielin gene cluster consists of seven genes (*FSL1*-*7*, Table 1) in *F. graminearum* of which *FSL1-5* and *FSL7* are predicted to be involved in fusarielin biosynthesis as they are conserved in *Aspergillus*, where several strain can produce fusarielins A and B [12,13].

The gene cluster is also preserved in species of *Metarhizium* [12], *Penicillium*, and *Colletotrichum* (Supplementary Table S1). Besides the PKS (*FSL1*), the remaining genes are predicted to encode a thioesterase (*FSL2*), an epimerase (*FSL3*), a cytochrome P450 oxygenase (*FSL4*), an enoyl reductase (*FSL5*), and a transcription factor (*FSL7*). The proposed *F. graminearum* cluster differs from *Aspergillus* by the presence of a gene encoding an AMP binding enzyme (*FSL6*). Transcription data suggests, furthermore, that this gene is not part of the biosynthetic pathway, as it is not co-expressed with the remaining cluster [12].

To gain insights into the biosynthetic pathway of the fusarielins in *F. graminearum*, we applied a knock out strategy where *FSL1*-*5* were targeted individually (Supplementary Figure S1) in a mutant constitutively overexpressing the local transcription factor *FSL7* [12]. *FSL6* was excluded from the study as it is unlikely to be part of the conserved fusarielin gene cluster [12]. The generated mutants were screened by diagnostic PCR and single integration was verified by Southern blot analyses. An additional larger band (approx. 6.5 kbp) was observed in the Southern blot analysis for OE::*FSL7*Δ*FSL1*, which could be caused by double integration or partial restriction enzyme digest.

**Figure 2:**
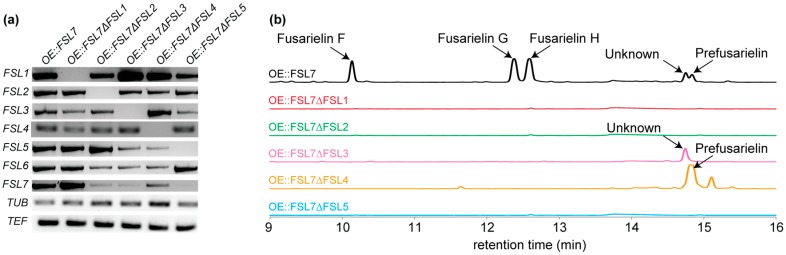
Transcription and metabolite analyses of OE::*FSL7* and OE::*FSL7*Δ*FSL1*-*5*. (**a**) Reverse transcription polymerase chain reaction (RT-PCR) of *FSL1*-*7*, the translation elongation factor 1α (TEF) and β-tubulin (TUB) were included as controls; (**b**) Partial HPLC-UV chromatograms measured at 249 nm.

The transcription profiles of representatives of the resulting mutants showed that deletion of the individual genes did not influence transcription of the remaining cluster as they were all detected in the respective transformants (Figure 2a). The metabolite profiles showed that the OE::*FSL7* mutant produced fusarielins F, G, and H as well as two additional hypothetically fusarielins (Figure 2b). These hypothetical fusarielins have been noticed previously and have identical masses corresponding to the chemical formula C_25_H_36_O_3_ [12]. As expected, fusarielin production was absent in the OE::*FSL7*Δ*FSL1* transformant lacking the PKS, but the loss of the putative thioesterase *FSL2* and of the enoyl reductase *FLS5* also abolished fusarielin production.

Production of fusarielins F, G, and H was absent in the OE::*FSL7*Δ*FSL3* mutant, which instead accumulated one of the hypothetical fusarielins (retention time: 14.7 min) with the chemical formula C_25_H_36_O_3_. Repeated attempts failed to elucidate the structure, as NMR data were inconclusive. This could be due to instability of the compound. Small amounts of this unresolved compound and another unknown compound (retention time: 15.1 min) were present in the OE::*FSL7*Δ*FSL4*, which accumulated the second hypothetical fusarielin in high levels (retention time: 14.8 min). The structure of this compound was elucidated based on 1D and 2D homo- and heteronuclear NMR experiments (Table 2).

The ^1^H NMR spectra suggested four olefinic hydrogens, which is one more than the three observed in fusarielins A–H [11,12]. The ^13^C NMR spectrum contained 25 resonances found from the compound corresponding to 6 methyl groups, 12 methines, 2 methylenes, and 5 quaternary carbon atoms. Of the quaternary carbon atoms, one was a carboxylic acid and the rest alkenes. The structure of this compound is similar to fusarielins F and G, with the exception that it contains a double bond between C15 and C16 instead of the monooxygenated methine (C16) and the keto group (C15) in fusarielin F or the epoxidation between C15 and C16 in fusarielin G [12].

**Figure 3:**
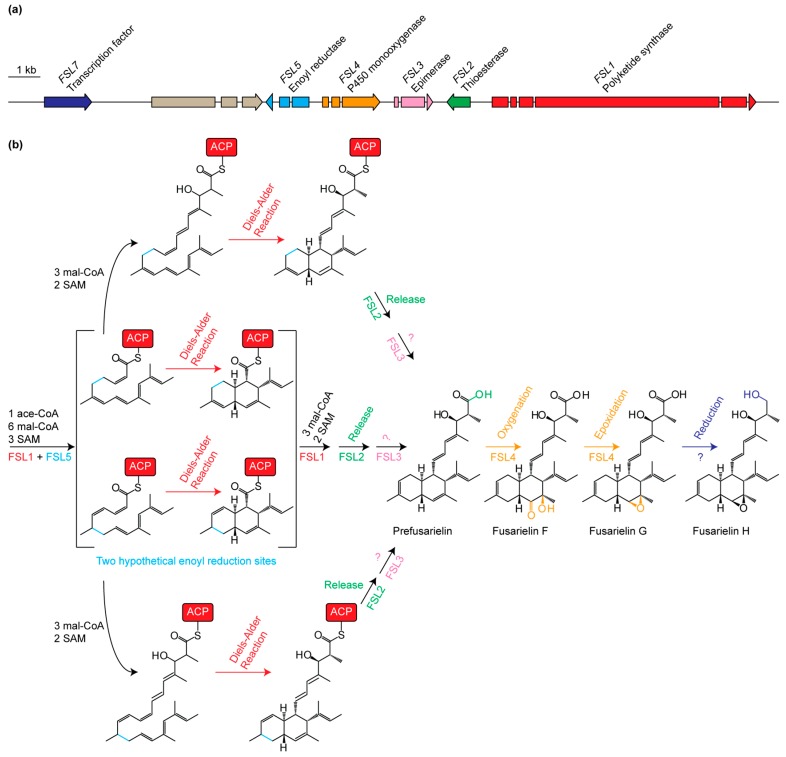
(**a**) Overview of the gene cluster with the six genes involved in fusarielin biosynthesis; (**b**) Possible pathways for fusarielin biosynthesis in *Fusarium graminearum*, which uses one acetyl-CoA (ace-CoA), nine malonyl-CoA (mal-CoA), and five S-Adenosylmethionine (SAM) subunits. The Diels-Alder reaction can occur at the heptaketide stage or when chain elongation has completed. The growing polyketide chain is attached to the acyl carrier protein (ACP) domain of FSL1. Two possible enoyl reduction sites are highlighted.

The metabolite data were furthermore used to propose a model for fusarielin biosynthesis in *F. graminearum* (Figure 3) based on models for lovastatin biosynthesis [3,10,15]. Analyses of the fusarielin and lovastatin gene clusters showed that there is some sequence similarity for the PKS’s (38% identity), *trans*-thioesterases (28% identity), and *trans*-ERs (46% identity), with E values from blastP analyses ranging from 0.0 to 1 × 10^−8^ (Supplementary Table S1).

The initial compound in the pathway is produced by FSL1, which is predicted to contain a β-ketosynthase, acyl-transferase, DH, methyltransferase, KR, and an acyl-carrier protein domain [12]. FSL1 contains an inactive ER domain [8] and biosynthesis of fusarielins relies on the predicted *trans*-acting ER FSL5, before it is released through hydrolysis catalyzed by FSL2. This suggest that there are several similarities to the biosynthetic pathway of lovastatin in which the polyketide chain is reduced at five sites by a *trans*-ER (lovC) [6,7] before it is released from the PKS by a trans-thioesterase (LovG) [10]. The Diels-Alder reaction is proposed to occur at the pentaketide stage in lovastatin biosynthesis, and it is therefore possible that fusarielin biosynthesis follows a similar route. Fusarielin biosynthesis could, however, also follow the betaenone pathway, where the Diels-Alder reaction is proposed to occur when chain synthesis is completed, either spontaneously or guided by the PKS [8].

Fusarielins F, G, and H have a C11=C12 *cis* double bond and is fully reduced between C10 and C11 and between C12 and C13. The *cis* double bond could arise from the Diels-Alder reaction or from the actions of enoyl-isomerase or reduction domains in modular PKS’s during chain elongation [16,17,18]. The *trans*-to-*cis* isomerization can also be catalyzed by exogenous post PKS tailoring enzymes, as observed in phoslactomycin biosynthesis in *Streptomyces*, which is catalyzed by an enzyme with homology to a nicotinamide adenine dinucleotide (NAD)-dependent epimerase/dehydratase [19]. FSL3 can play a similar role in fusarielin biosynthesis to form the C11=C12 *cis* double bond by moving a hypothetical C10=C11 or C12=C13 *trans* double bond. Recently, fusarielin I was identified in a marine *Penicillium* strain, which differs from the known fusarielins in the absence of a C11=C12 *cis* double bond [20]. This indicates enoyl reductions at C10=C11 and C12=C13 in this fungus and future analyses can shed light on how the gene cluster is organized.

In *F. graminearum*, prefusarielin is oxygenated at C15 and C16 by FSL4, resulting in fusarielin F, which subsequently is epoxidized into fusarielin G by the same enzyme. To gather further evidence for the role of FSL4 in oxygenation of C15 and C16, the binding affinity of FSL4 to fusarielins was investigated through in silico docking analyses. Binding analyses confirmed furthermore that C15 and C16 is the likely site of action for FSL4 as this area is the vicinity of the heme-binding site (Supplementary Figure S2). The final step in the pathway is a reduction of the carboxylic acid moiety by a mechanism which could not be determined with the available data.

## 3. Materials and Methods

### 3.1. Generation of FSL1-5 Knock Out Mutants

Border regions (435–1120 bp) of *FSL1*-*5* were amplified by PCR using the primers listed in Supplementary Table S2. The PCR was performed with PfuTurbo Cx Hotstart DNA polymerase (Stratagene, La Jolla, CA, USA) on genomic DNA from *F. graminearum* PH-1 (NRRL 31084). The border regions were cloned into a linearized GU2 vector carrying a G418 resistance cassette [21] by a four fragment cloning step using the USER enzyme (New England Biolabs, Ipswich, MA, USA) [22] and verified by colony PCR and subsequent sequencing of the integration sites by Eurofins Genomics (Ebersberg, Germany). The verified vectors were electroporated into *Agrobacterium tumefaciens* and used to transform conidia of an overexpression mutant of *FSL7* [12], as described previously [23]. The conidia has been obtained from a four-day-old culture grown in liquid sporulation medium [24] at 20 °C with 100 rpm. The knock out mutants were selected on defined *Fusarium* medium (DFM; [22]) containing 300 μg/mL of G418. Correct integration of the knock out cassettes was verified by diagnostic PCR using forward primers (Supplementary Table S2) annealing outside the integration site in combination with a reverse primer annealing to the G418 resistance cassette (Supplementary Figure S1). Single event integration was verified by Southern blot analyses using genomic DNA digested with *Kpn*I (Δ*FSL1*, *2*, *4* and *5*) and *Bam*HI (Δ*FSL3*) and hybridized with a probe specific for the G418 resistance cassette generated with primers P300 and P301 (Supplementary Figure S1).

### 3.2. Transcription Analyses

Mutant strains carrying representative knock out cassettes of each gene were chosen for further analyses. The strains were grown on solid yeast extract sucrose (YES; [25]) medium (Scharlau, Barcelona, Spain) for two weeks in the dark at 25 °C. Aerial mycelium was lyophilized and pulverized by the addition of beads which were shaken at 2 × 10 s intervals in a bead beater. The RNA was then extracted using the RNeasy plant mini kit (Qiagen, Hilden, Germany) according to the manufacturer’s instructions. The RNA quality was checked by agarose gel electrophoresis, and first stand synthesis was performed with oligo (dT) primers using the SuperScript III reverse transcriptase (Invitrogen Life Technologies, Carlsbad, CA, USA). The RT-PCR was performed using the Paq500 polymerase (Stratagene). Two housekeeping genes—*β-tubulin* (FGSG_06611) and *translation elongation factor 1α* (FGSG_08811)—were included as controls.

### 3.3. Metabolite Profiling

Nine plugs were taken from each of the YES cultures and extracted ultrasonically for 45 min with 1.5 mL of ethyl acetate/dichloromethane/methanol (3:2:1) containing 1% formic acid [26]. The samples were lyophilized and re-dissolved ultrasonically for 10 min in 600 μL of methanol. Impurities were pelleted by centrifugation at 14,000 rcf for 1.5 min before the extracts were transferred to 2 mL high-performance liquid chromatography (HPLC) vials. The extracts were analyzed on an Agilent 1260 LC system (Agilent Technologies, Waldbronn, Germany) equipped with a diode array detector collecting spectra between 200 and 600 nm. One microliter extract was injected and separated on a 100 × 2.1 mm kinetex 2.6 μm phenyl-hexyl (Phenomenex, Torrance, CA, USA) using a flow of 0.4 mL/min with a linear water–acetonitrile gradient, where both eluents were buffered with 50 ppb trifluoroacetic acid. The gradient started at 10% acetonitrile and reached 100% in 12 min, which was held for 3 min.

### 3.4. Isolation and Elucidation of Prefusarielin

OE::*FSL7*Δ*FSL4* was grown on 50 Petri dishes with YES medium for two weeks in the dark at 25 °C. The agar plates were cut in 0.5 × 0.5 cm squares and divided into two 1 L bottles and extracted ultrasonically with 1 L of ethyl acetate for 45 min. The extract was filtered through miracloth and evaporated to dryness. The extracts where then re-dissolved in 100 mL of methanol, and prefusarielin was isolated by multiple cycles on an Agilent 1260 semi-preparative HPLC system equipped with a 150 × 10 mm Gemini 5 μm C6-Phenyl 110 Å column (Phenomenex, Torrance, CA, USA) using a flow of 5 mL/min with a linear water-acetonitrile gradient, where both were buffered with 50 ppm trifluoroacetic acid. The gradient started at 50% acetonitrile and reached 100% in 10 min, which were held for 2 min before reverting to 50% acetonitrile.

The structure of prefusarielin was elucidated using NMR on a Bruker AVIII-600 spectrometer (Bruker, Karlsruhe, Germany). Approximately 3 mg of the compound were dissolved in 550 μL DMSO-*d*_6_ and analyzed with ^1^H, ^13^C, 2D-[^1^H-^13^C]-HSQC, HMBC, 2D-[^1^H-^1^H]-COSY, 2D-ROESY, and 2D-DOSY at 308 K. Spectra were recorded and analyzed with TopSpin 3.2 (Bruker, Karlsruhe, Germany). All chemical shifts are relative to internal tetramethylsilane (TMS).

### 3.5. Docking Analyses of FSL4

An initial model of FLS4 (residues 58–554) was generated in SWISS-MODEL [27] (Basel, Switzerland; accessed 23 July 2016), using PDB ID 4D6Z as the template. Following coordination of oxy-heme or deoxy-heme groups, further energy minimization and model refinement during a 500 ns molecular dynamics simulation was conducted in YASARA/WHAT IF Twinset [28,29] with the Yasara2 forcefield and explicit water (TIP3P water model) [30] (Vienna, Austria; version 16.7.22). Using the best Z-scoring conformer [31], energy-minimized prefusarielin or fusarielin F ligands were docked globally in AutoDock VINA (500 docking runs, Yasara2 charge assignment) [32]. Evaluation was based on two criteria, with criteria one and two weighing the distance of the C15-C16 ligand bond to the heme-group (shorter is better) and the binding affinity (higher is better), respectively.

## 4. Conclusions

Through targeted deletion of genes in the fusarielin gene cluster, we were able to propose a model for fusarielin biosynthesis in *F. graminearum*. The initial compound in the pathway is produced by the synthase FSL1 in collaboration with the *trans*-acting enoyl reductase FSL5 and the thioesterase FSL2. The fusarielins produced by *F. graminearum* have a C11=C12 *cis* double bond, which could result from the Diels-Alder reaction or from the action of FSL3 in a *trans*-to-*cis* isomerization event. This compound is then oxygenized to fusarielin F and epoxidized to fusarielin G before the carboxylic acid moiety is reduced to alcohol in fusarielin H.

## Figures and Tables

**Table 1:** Genes in the fusarielin (*FSL*) gene cluster and their proposed function.

Gene Name (Accession Number) [14]	Protein Family	Proposed Function
*FSL1* (FGSG_10464)	Polyketide synthase	Reducing PKS; condensation of ten acetate units
*FSL2* (FGSG_10463)	Thioesterase	Release of product from FSL1
*FSL3* (FGSG_17368)	Aldose 1-epimerase	Epimerization of unknown entry product
*FSL4* (FGSG_10461)	Cytochrome P450	Oxygenation of C-15 and C-16
*FSL5* (FGSG_17367)	Enoyl reductase	Enoyl reduction at C10=C11 or C12=C13
*FSL6* (FGSG_10459)	AMP-binding	Unknown role or not involved in biosynthesis
*FSL7* (FGSG_10458)	Transcription factor	Transcriptional regulation of the fusarielin gene cluster

AMP: adenosine monophosphate; PKS: polyketide synthase.

**Table 2:** NMR spectroscopic data (600 MHz, DMSO-*d*_6_) and structure of prefusarielin.

**Pos.**	**^1^H Shift (ppm) ^1^**	**^13^C Shift (ppm)**	**Pos.**	**^1^H Shift (ppm) ^1^**	**^13^C Shift (ppm)**	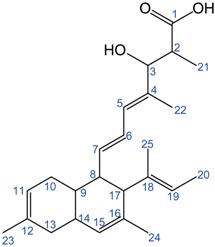
1		176.40	13	1.69, 1H, dd	37.46	
2	2.38, 1H, dq	43.53		1.99, 1H, dd		
3	3.92, 1H, d	78.80	14	1.90, 1H, dddd	38.06	
4		135.53	15	5.36, 1H, s	126.62	
5	5.90, 1H, d	126.89	16		134.05	
6	5.32, 1H, d	136.34	17	2.58, 1H, d	54.10	
7	6.24, 1H, dd	126.09	18		134.60	
8	2.21, 1H, ddd	47.73	19	5.16, 1H, q	122.07	
9	1.41, 1H, dddd	34.02	20	1.57, 3H, d	13.38	
10	1.44, 1H, ddd	30.91	21	0.81, 3H, d	13.99	
	1.82, 1H, ddd		22	1.62, 3H, s	11.01	
11	5.34, 1H, dd	121.31	23	1.62, 3H, s	23.12	
12		133.31	24	1.54, 3H, s	18.01	
			25	1.51, 3H, s	21.88	

^1^ Multiplicity of signals: s, singlet; d, doublet; q, quartet.

## Supplementary Materials

Supplementary materials can be accessed at: http://www.mdpi.com/1420-3049/21/12/1710/s1.
