# Supplementary material for: Functional Analysis of the Fusarielin Biosynthetic Gene Cluster
Source: Molecules. 2016 Dec 13;21(12):1710. doi: 10.3390/molecules21121710 (PMC6274466; doi:10.3390/molecules21121710)
Supplement: Supplementary file 1 [file molecules-21-01710-s001.pdf]

Supplementary Materials: Functional Analysis of the Fusarielin Biosynthetic Gene Cluster

Aida Droce, Wagma Saei, Simon Hartung Jørgensen, Reinhard Wimmer, Henriette Giese, Rasmus Dam Wollenberg, Teis Esben Sondergaard and Jens Laurids Sørensen

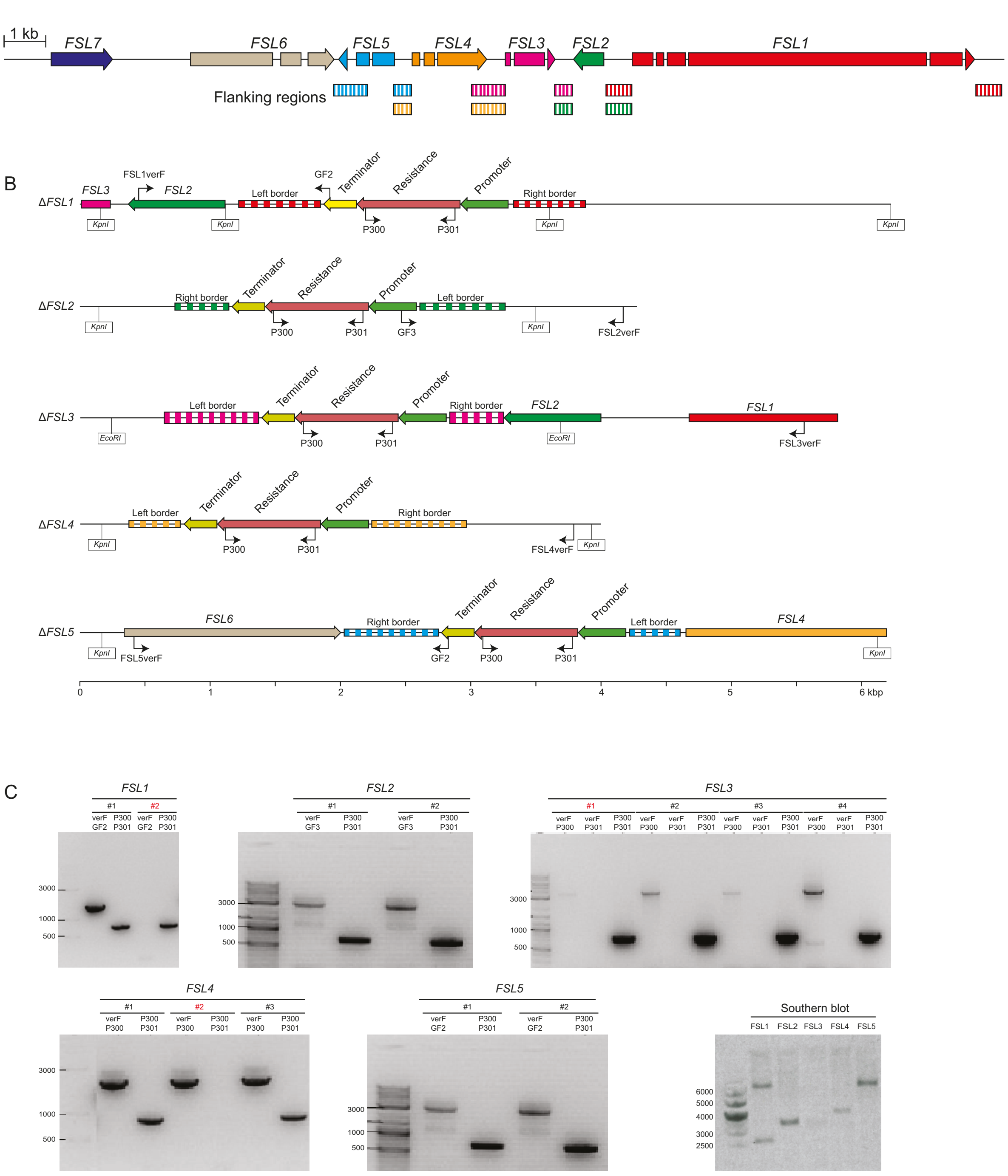

Figure S1. Generation and verification of deletion mutants. (**A**) Overview of the fusarielin gene cluster with highlighted border regions used for generation of knock-out vectors; (**B**) Illustration of the genomic DNA regions for knock-out mutants of *FSL1-5* showing the border regions, geneticin resistance cassette, primer sites used for verification and restriction sites (*KpnI* and *EcoRI*, respectively) used for Southern blot analyses; (**C**) Verification of mutants with PCR and Southern blotting. Incorrect mutants are labelled in red.

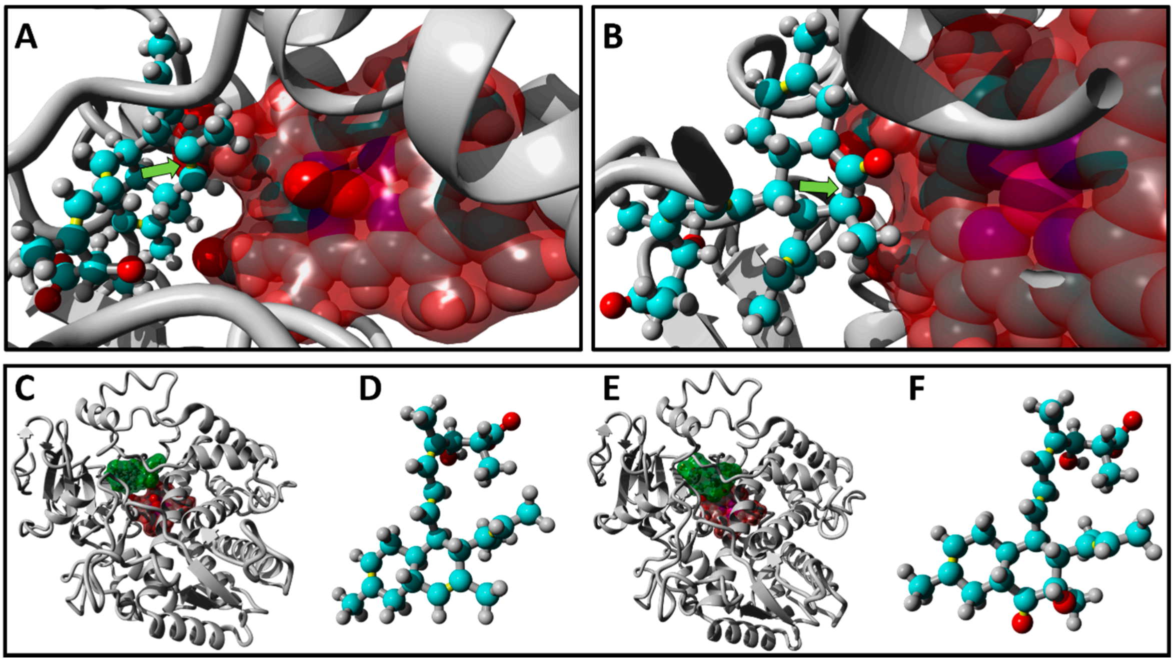

Figure S2. Docking of prefusarielin and fusarielin F into energy-minimized homology models of FSL4 (grey ribbon diagrams). (**A**) A prefusarielin docking pose near the oxy-heme group (red surface-rendering) bound within the oxy-heme FSL4 model. The green arrow indicates the position of the C15-C16 double-bond in prefusarielin; (**B**) A similar docking-pose of fusarielin F near the deoxy-heme group, with the green arrow indicating the position of the C15-C16 double-bond; (**C**) Global view of the prefusarielin docking pose (surface-rendered green) within the oxy-heme FSL4 homology model; (**D**) Energy-minimized prefusarielin ligand; (**E**) Global view of the fusarielin F docking-pose (surface-rendered green) within the deoxy-heme FSL4 homology model; (**F**) Energy-minimized fusarielin F ligand.

Table S1. Conservation of the fusarielin gene cluster in *Aspergillus clavatus*, *Penicillium nordicum* and *Colletotrichum simmondsii* and comparison to lovastatin gene cluster in *Aspergillus terreus* [6,7,10]. Numbers in parentheses are E values from BlastP analyses.

Gene Name	Length (bp/aa)	*Aspergillus clavatus*	*Penicillium nordicum*	*Colletotrichum simmondsii*	*Aspergillus terreus*
FSL1 (FGSG_10464)	8224/2642	ACLA_098920 (0.0)	ACN38_g5709 (0.0)	CSIM01_02879 (0.0)	LovB; ATEG_09961 (0.0)
FSL2 (FGSG_10463)	747/248	ACLA_098880 (1e-61)	ACN38_g5705 (8e-69)	CSIM01_02875 (5e-66)	LovG; ATEG_09962 (1e-08)
FSL3 (FGSG_17368)	1215/362	ACLA_098890 (1e-146)	ACN38_g5705 (1e-138)	CSIM01_02876 (5e-66)	
FSL4 (FGSG_10461)	1808/557	ACLA_098900 (2e-164)	ACN38_g5707 (2e-163)	CSIM01_02878 (7e-165)	LovA; Q9Y7C8.1 (no hit)
FSL5 (FGSG_17367)	1279/299	ACLA_098910 (5e-151)	ACN38_g5708 (8e-147)	CSIM01_02877 (5e-138)	LovC; ATEG_09963 (1e-82)
FSL6 (FGSG_10459)	3460/1045	-	-	-	
FSL7 (FGSG_10458)	1491/496	ACLA_098870 (0.02)	ACN38_g5704 (9e-09)	CSIM01_02875 (2e-14)	

Table S2. Primers used in the study to create knock-out vectors, verify *F. graminearum* mutants and for RT-PCR.

Name	Sequence	Amplicon
FSL5-A1<br>FSL5-A2	5′-GGTCTTAAXCTTGAAGCTTAGAGGCT<br>5′-GGCATTAAXCTTGAGGGAGCTGAG	418 bp
FSL5-A3<br>FSL5-A4	5′-GGACTTAAXCACACGCAACTCACTCA<br>5′-GGGTTTAAXGGAGGATGAGGTCTTG	736 bp
FSL4-A1<br>FSL4-A2	5′-GGTCTTAAXCTTGAGGGAGCTGAGTA<br>5′-GGCATTAAXCTTGAAGCTTAGAGGCTG	402 bp
FSL4-A3<br>FSL4-A4	5′-GGACTTAAXCACCCAGCGCCG<br>5′-GGGTTTAAXGGTATGACGACGGGTA	729 bp
FSL3-A1<br>FSL3-A2	5′-GGTCTTAAXCACCCAGCGCCG<br>5′-GGCATTAAXGGTATGACGACGGGTA	745 bp
FSL3-A3<br>FSL3-A4	5′-GGACTTAAXGCTGGATCGCCATAAG<br>5′-GGGTTTAAXAGATCACTGGTTTCAAGCA	435 bp
FSL2-A1<br>FSL2-A2	5′-GGTCTTAAXGATGAATTATAATGTATTTCTGAATGAAT<br>5′-GGCATTAAXTTCTGTGCTTGTGATTGTTTC	660 bp
FSL2-A3<br>FSL2-A4	5′-GGACTTAAXAGATCACTGGTTTCAAGCA<br>5′-GGGTTTAAXGCTGGATCGCCATAAG	419 bp
FSL1-A1<br>FSL1-A2	5′-GGTCTTAAXTTCTGTGCTTGTGATTGTTTC<br>5′-GGCATTAAXGATGAATTATAATGTATTTCTGAATGAATG	660 bp
FSL1-A3<br>FSL1-A4	5′-GGACTTAAXGGAAAGACAAGAGCAGG<br>5′-GGGTTTAAXGTTGATAATTGTAAAAGCGATATATG	587 bp
FSL5-verF	5′-TTCTTTGTACACAGGCCGACAGGACGCAATGA	2418 bp (in combination with GF2)
FSL4-verF	5′-GATGCAACTTGTGGTCGCGTACCGTTTTATCACT	2691 bp (in combination with P300)
FSL3-verF	5′-TCCTCAATAGCTGCACTGAGTGGCTTCA	3848 bp (in combination with P300)
FSL2-verF	5′-TCCTCAATAGCTGCACTGAGTGGCTTCA	1693 bp (in combination with GF3)
FSL1-verF	5′-CATTATAGCAGACCAGGCATCTTCTA	1472 bp (in combination with GF2)
GF2-verR	5′-GTTGTTGACCCCTATACTCTT	
GF3-verR	5′-AGACCCAATTACACCCTT	
P300<br>P301	5′-GTAAAGCACGAGGAAGCG<br>5′-TTGGGTGGAGAGGCTATT	690 bp (verification of geneticin)
FSL7-RT292<br>FSL7-RT299	5′-ACAGCGACGCTAAGCTTCGAGCCGCCTGCGATA<br>5′-TCGCATCAACAGTTGAATATAAACACCGTA	717 bp
FSL6-RT270<br>FSL6-RT272	5′-CCAACACTATTTTACTCACTGGCGCCA<br>5′-TACCGCCGTTCCGCGATATTGCCCTT	503 bp
FSL5-RT296<br>FSL5-RT297	5′-ATAGACGGCAAAATGGTTGGCAATCTGGCTA<br>5′-AACATGTGGTGATGGGGATCAACCCGGAGAGT	409 bp
FSL4-RT273<br>FSL4-RT275	5′-GCCTTTTCGCCATCTTCCAACACCCGCGAGCCGTTCA<br>5′-GATTGGATTTCCCAAAGTCATGAGAATGGTTA	191 bp
FSL3-RT294<br>FSL3-RT295	5′-AATCCTTACCTGAACGGTGCTATTGGTCGATA<br>5′-ATGCAACTTGTGGTCGCGTACCGTTTTATCACT	422 bp
FSL2-RT276<br>FSL2-RT277	5′-TTCAAAGTACTGCGAATCATCAAGACGGATA<br>5′-TGCGCAGAGTCCCAACATGAAAGCACAG	701 bp
FSL1-RT278<br>FSL1-RT280	5′-CAATTGGCATGGTCCCTCAATGACCATTGA<br>5′-TCCTCAATAGCTGCACTGAGTGGCTTCA	282 bp
TEF1-RT152<br>TEF1-RT153	5′-GGAGGAGAAGACTCACCTTAACGTCG<br>5′-CGGTGACATAGTAGCGAGGAGTCTC	260 bp
TUB-RT150<br>TUB-RT151	5′-TGACTGGTCGCAACATGAAGACTTTCC<br>5′-GCGGTGGAGTTGCCAACGAAA	301 bp
